# Increased Posterior Condylar Offset Decreases the Extension Gap in Cases With Flexion Contracture in Modified Gap Technique Total Knee Arthroplasty

**DOI:** 10.7759/cureus.59067

**Published:** 2024-04-26

**Authors:** Goki Kamei, Akinori Nekomoto, Yu Mochizuki, Masakazu Ishikawa, Nobuo Adachi

**Affiliations:** 1 Department of Orthopedic Surgery, Graduate School of Biomedical and Health Sciences, Hiroshima University, Hiroshima, JPN; 2 Department of Orthopedic Surgery, Hiroshima Prefectural Hospital, Hiroshima, JPN; 3 Department of Orthopedic Surgery, Faculty of Medicine, Kagawa University, Kagawa, JPN

**Keywords:** posterior condylar offset, modified gap technique, posterior stabilized total knee replacement, total knee arthroplasty technique, total knee arthroplasty (tka)

## Abstract

Purpose

There have been no reports comparing the change in medial and lateral posterior condylar offset (PCO) and the extension gaps. The purpose of this study was to elucidate the relationship between the change in medial and lateral PCO and the extension gap in total knee arthroplasty (TKA). The hypothesis is that an increase in both medial and lateral PCO can be a factor for a decrease in the extension gap, especially in cases of flexion contracture.

Methods

This retrospective study included 63 patients with medial osteoarthritis who underwent mobile-bearing PS-TKA using the modified gap techniques. Patients consisted of seven men (seven knees) and 53 women (56 knees), with the mean age of 76 (range, 58-88) years. The patients with valgus knee and cruciate retaining TKA were excluded. The medial ΔPCO (ΔPCO defined as the amount of change of the PCO before the resection of the posterior condyle and after the implant setting), lateral ΔPCO, the rotation angle of the posterior condyle osteotomy, and the gap differences were evaluated. The data were compared among three groups（Group A: ΔPCO increase on both sides, Group B: ΔPCO increase on only one side, Group C: ΔPCO decrease on both sides. The gap differences were compared between the cases with flexion contracture of ≥ 15° and the cases with flexion contracture of < 15°. The correlations between the gap differences and flexion contracture were evaluated in each group.

Results

There was no gap difference evident in any group (P≥0.05). The gap difference in Groups A (P=0.0067) and group C (P=0.0484) was significantly larger in cases with flexion contracture of ≥ 15° compared to those with flexion contracture of < 15°.

Conclusions

There was no correlation between the change in PCO and the extension gap. However, there was an inverse correlation between the flexion contracture and extension gap in cases with increased medial and lateral PCO.

## Introduction

In modified gap technique total knee arthroplasty (TKA), the extension and flexion soft tissue balance determines the rotation angle and the antero-posterior position (where the femoral component is placed) [1-4]. Therefore, posterior condylar offset (PCO) may increase either medially or laterally, both medially and laterally, or decrease both medially and laterally. Regarding the change in the PCO and the extension gap, it has been reported that an increase in PCO decreases the extension gap [5-7]. We needed to consider how the extension gap is affected in each case. The association between the gap difference and preoperative flexion contracture is also examined because preoperative flexion contracture is a factor affecting the intraoperative and postoperative extension gap [8]. No research has yet compared the changes in PCO and extension gap, evaluated before the resection of the posterior condyle with the distal femoral trial and after the resection of the posterior condyle with the placement of the femoral trial component [9,10]. The purpose of this study was to elucidate the relationship between the change in medial and lateral PCO and the extension gap using a TKA modified-gap technique. The effect of flexion contracture on the extension gap was also examined. The hypothesis is that an increase in both medial and lateral PCO can be a factor for a decrease in the extension gap, especially in cases of flexion contracture.

## Materials and methods

**Figure 1:**
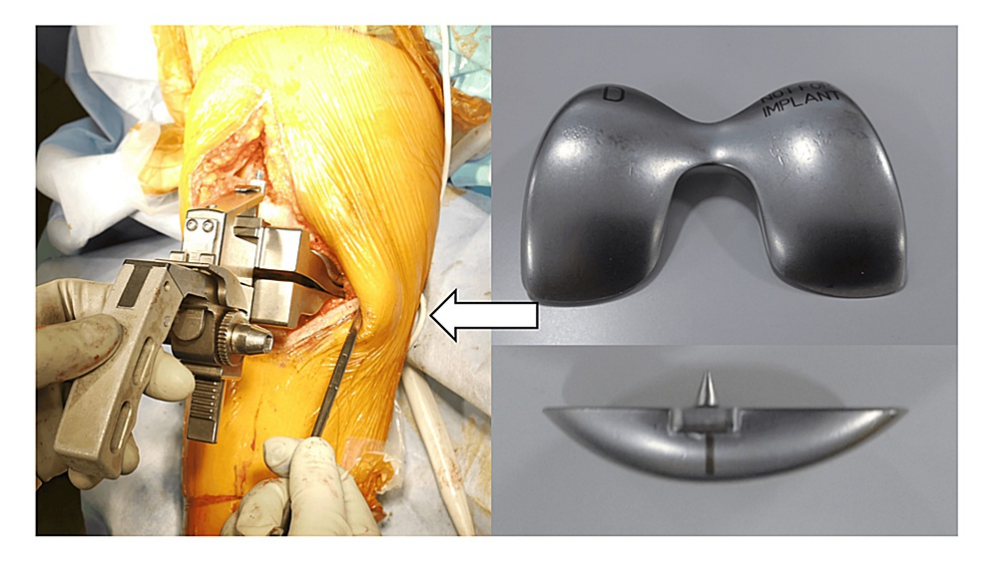
A distal femoral trial. The extension gap was measured by inserting a distal femoral trial.

TKA was performed for 96 patients between May 2017 and December 2018. This retrospective study included 63 patients with medial osteoarthritis who underwent mobile-bearing posterior-stabilized (PS)-TKA (Zimmer-Biomet’s PSRP Knee System, Warsaw Indiana, USA) using the modified gap techniques. It was possible to compare the extent of osteotomy of the posterior condyle and the extension gap (before posterior condyle resection) with the distal femoral trial (Figure 1), the extension gap (after posterior condylar resection), and the femoral component.

The demographics and clinical features of all included participants are presented in Table 1. Exclusion criteria were (1) valgus knee, the percentage of mechanical axis (%MA) more than 50％, and (2) cases using other implants. The flow chart of included patients is shown in Figure 1. Informed consent was obtained from all participants. All procedures were performed using the modified gap techniques by a single surgeon (G.K.).

**Table 1:** Data of three groups according to ΔPCO. Group A: Increase on both sides; Group B: Increase on only one side, Group C: Decrease on both sides. ΔPCO is defined as the amount of change of the posterior condylar offset (PCO) before the resection of the posterior condyle and after the implant setting.

	Group A	Group B	Group C	P value
Medial ΔPCO (mm)	1.59±1.30	-1.50±1.52	-3.67±1.41	P＜0.01
Lateral ΔPCO (mm)	1.38±0.76	0.79±1.80	-1.81±1.56	P＜0.01
Rotation angle of posterior condylar osteotomy（°）	3.31±2.09	4.11±1.85	3.14±1.27	0.0450
Gap difference (mm)	1.63±2.33	1.79±2.45	1.14±2.41	0.3525
Gap difference (mm) preoperative extension limitation of 15° or more	3.0±1.58	1.68±2.15	2.5±2.88	0.2711

There were seven men (seven knees) and 53 women (56 knees) with a mean age of 76 years (range: 59 ～ 89 years). The preoperative mean range of motion was -11.3° ± 0.9° (range: -25° ～ 0°) in extension and 109.6° ± 2° (range: 70° ～ 135°) in flexion. The ％MA** **was 0.23±23.0% (range: -66.0%～40％). In the Kellgren-Lawrence stage, there were five cases in grade 2, 21 cases in grade 3, and 37 cases in grade 4. All data were retrospectively corrected and analyzed from an institutional database (Hiroshima University Hospital and Hiroshima Prefectural Hospital). This study was approved by our institutional review board (Hiroshima University, E-2774).

Surgical technique

The following methods were performed in all cases. The knees were exposed via the medial parapatellar approach, and the patella did not resurface in all patients. Both anterior and posterior cruciate ligaments were resected. Then, the distal femur was resected perpendicular to the mechanical axis using an intramedullary guide (concerning the angle between the anatomic and mechanical axes of the femur). The tibia was resected perpendicular to the tibial mechanical axis using an extramedullary guide and was dissected 10 mm from the lateral articular surface of the tibia. If a sufficient extension gap cannot be obtained such that the thinnest insert cannot be inserted, an additional osteotomy of the tibia is performed. The extension balance was measured using a Stryker offset tensor (a JDK offset balancer; Stryker, Mahwah, NL) at 30 lbs. joint distraction after obtaining a proper soft tissue balance in knee extension [9]. A proper soft tissue balance was defined as an intraoperative joint gap inclination of 0° to 3°. Osteotomy of the posterior femoral condyle was performed so that the extension gap and flexion gap had the same value, and the flexion gap inclination was 0°.

Gap measurement

**Figure 2:**
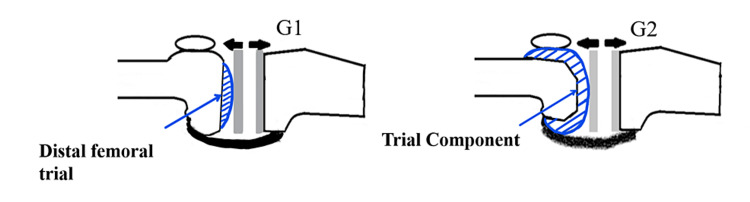
Gap difference. G1 (distal trial gap): the extension gap before posterior condyle resection. G2 (component gap): the extension gap after posterior condyle resection. The gap difference was defined as G1-G2.

The extension gap (before posterior condyle resection: distal trial gap: G1) was measured by inserting a distal trial after adjustment of the soft tissue balance [10]. The extension gap (after posterior condyle resection: component gap: G2) was measured after both the posterior condyle resection and insertion of the femoral trial component. The difference between the distal trial gap (G1) and the component gap (G2) was defined as the gap difference (G1 - G2) (Figure 2).

Change in PCO

The extent of osteotomy of the medial and lateral posterior condyle was measured to the first decimal place using a caliper, and the value added to the thickness of the bone saw (1.27 mm) was defined as the actual amount of osteotomy. The implant thickness minus the actual osteotomy measurement was defined as the amount of change of the posterior condylar offset (ΔPCO).

Correlation between extension gap and change in PCO

Group A was classified as having an increase on both medial and lateral sides, group B as having an increase on only one side, and group C as having a decrease on both medial and lateral sides. The correlations between the medial ΔPCO or lateral ΔPCO and the extension gap difference were compared. The gap differences between the cases in the three groups with flexion contracture of ≥15° and those with flexion contracture of <15° were also evaluated: the definition of flexion contracture followed the criteria of Hiranaka et al. [8].

Statistical analysis

The three groups were compared using the Kruskal-Wallis test, and correlation coefficients were compared using Spearman's correlation. P <0.01 was considered to be significant. Statistical analysis was performed using the software Statistical Package for Social Sciences (SPSS), version 22.0 (IBM Corp., Armonk, NY).

## Results

Table 2 shows that Group A had eight knees, Group B had 27 knees, and Group C had 28 knees.

**Table 2:** Data of gap difference according to preoperative restrictions of knee extension. The number of knees is in parentheses.

	Flexion contracture＜15	Flexion contracture 15≤	P value
Group A	-0.67±1.15 (3)	3.0±1.58 (5)	0.0067
Group B	2.12±2.80 (15)	1.38±2.31 (12)	0.4225
Group C	0.76±2.26 (20)	2.5±2.88 (8)	0.0484

**Figure 3:**
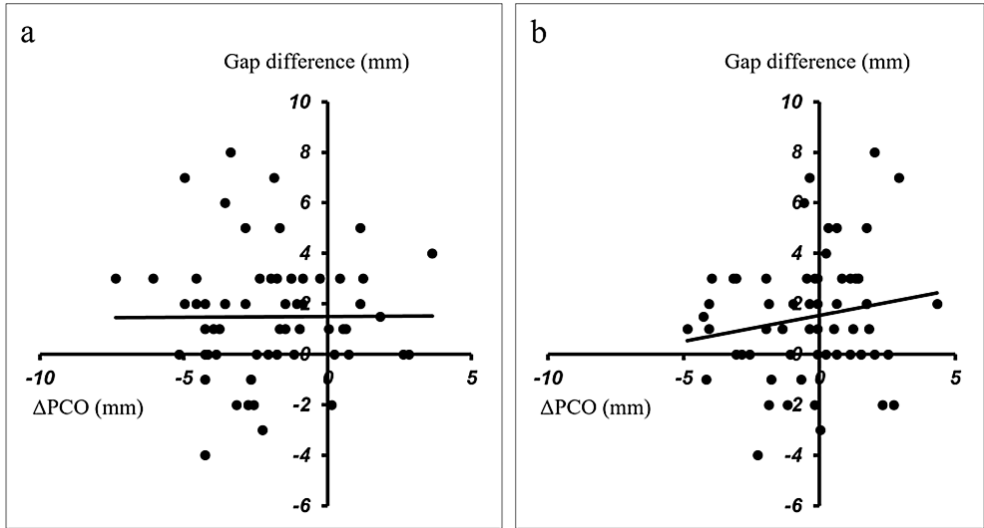
Correlation of the ΔPCO and the gap difference. a: There was no correlation between the medial ΔPCO and the gap difference (R=0.0393, P=0.3797). b: There was no correlation between the lateral ΔPCO and the gap difference (R=0.1368, P=0.1426). ΔPCO is defined as the amount of change of the posterior condylar offset (PCO) before the resection of the posterior condyle and after the implant setting.

There were statistically significant differences in medial ΔPCO, lateral ΔPCO, and the rotation angle of the posterior condyle osteotomy among the three groups. No variation in the gap difference was evident in any group. There was no correlation between the medial ΔPCO and the gap difference (Figure 3A). There was a statistically weak correlation between the lateral ΔPCO and the gap difference (Figure 3B).

**Figure 4:**
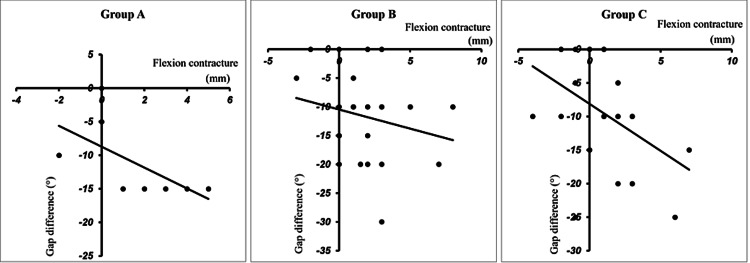
Correlation of the gap difference and flexion contracture. a:  Group A; there was a significant and strong correlation. (R=0.8457, P=0.0041). b:  Group B; there was no correlation. (R=0.1904, P=0.1708). c:  Group C; there was a significant and weak correlation. (R=0.3904, P=0.0200).

In Group A, there was a significantly larger gap difference between cases with preoperative flexion contractures of <15° and those with preoperative flexion contractures of ≥15°. There was no significant difference between cases with preoperative flexion contracture of <15° and those with preoperative flexion contracture of ≥15° in Groups B and C (Table 2). Group A had a significant and strong correlation between the gap difference and flexion contracture (Figure 4).

## Discussion

The most important findings of this study were that the extension gap decreased in cases with flexion contracture if both medial and lateral PCO increased after the implant was set. Conversely, the change of PCO in the circumstances without flexion contracture did not affect the extension gap. The clinical implication of this study is that the influence of PCO on the extension gap can be evaluated in more detail by evaluating changes in medial and lateral PCO respectively, and the surgeon would be able to achieve appropriate soft tissue balance during TKA surgery by taking the results into consideration.

The changes in PCO have been reported to affect the extension gap, postoperative flexion range of motion, and patellar tracking [5-7, 11-16]. Increased PCO is expected to result in a smaller extension gap due to increased posterior soft tissue tension in the extension position, which causes postoperative knee flexion contracture. Onodera et al. reported that PCO after placement of the femoral implant was greater than that in a healthy knee, and excessive posterior offset caused flexion contracture [17]. The flexion gap is adjusted according to the extension gap, particularly in posterior-stabilized (PS)-TKA using the modified gap technique. Accordingly, in cases where the posterior condyle osteotomy is reduced, an increase in the PCO and a decrease in the extension gap are expected. Muratsu et al. noted that the joint gap was measured with 40 lb. of joint distraction before (bone gap) and after the femoral trial component (component gap), and the bone gap was 5.3 mm less than the component gap because the posterior condyle of the femoral component tightens the posterior capsule [6]. Tsubosaka et al. showed that the joint component gap at 0° extension inversely correlates with the PCO, and the difference of the joint component gap between 10° and 0° positively correlates with the PCO [7]. Berger et al. and Miller et al. indicated that the femoral component should be placed in the external rotation position rather than on the surgical epicondylar axis because the internal rotation of the femoral component can result in patellofemoral disorders [12,14]. Therefore, an increase in lateral PCO is expected in many cases of modified gap TKA. Furthermore, depending on the anterior and posterior position of the femoral component, it is expected that there will be cases in which both the medial and lateral PCO become larger, or only one side becomes larger, or both the medial and lateral PCO become smaller.

There have been no previous studies that have demonstrated a correlation between the change in PCO and the extension gap before resection of the posterior condyle and after placement of the femoral trial component. The present study evaluated the gap difference before osteotomy of the posterior condyle and after placement of the femoral component in the same condition. This was because we thought the effects of changes in PCO on the extension gap might differ from group to group. In the present study, there was no correlation between the ΔPCO and gap difference among the three groups. Previous reports have compared the difference between the bone gap and the component gap, which do not have the same knee extension angle when the gap is measured. Conventional bone gaps are measured where the posterior condyle is absent, resulting in a large extension gap. Alternatively, the mean component gap for each study has been compared. The correlation between the PCO change and extension gap before and after posterior condylar osteotomy has not been examined in detail. This research compared the component gap (after posterior condyle osteotomy) with the distal femoral trial gap (before posterior condyle osteotomy) at almost the same angle of extension using the distal femoral trial, with gap values that could have been measured at nearly the same angles of extension. Moreover, the measurement was made after soft tissue balancing; therefore, the only significant extension gap factor was the change in PCO. This differs from previous studies in that the difference between the ΔPCO and the gap difference was not found in the present study [6,7].

Preoperative flexion contracture can be treated by additional osteotomy of the distal femur and release of the medial collateral ligament, posterior cruciate ligament, and other soft tissue procedures. If postoperative flexion contracture remains painful, edema and poor clinical outcomes can be expected, requiring revision surgery such as posterior capsular release and revised TKA [18-20]. Therefore, it is important to avoid postoperative flexion contracture. This study showed a strong inverse correlation between preoperative flexion contracture and intraoperative extension gap (component gap) in Group A, where PCO increased medially and laterally. Okamoto et al. reported that more than 1 mm laxity is necessary to avoid postoperative flexion contracture; flexion contracture occurred in five of 41 cases when the laxity is less than 1 mm [21]. However, they did not discuss the relationship between the PCO and flexion contracture. In the present research, a gap difference in patients with preoperative flexion contracture of ≥5° was 3.0 mm and was significantly larger (about 3.6 mm) than that of the patients with <15° in Group A. When both PCOs are expected to increase in patients with flexion contracture of ≥15°, we need to create a larger extension gap than in patients without flexion contracture.

Limitations

One limitation of this study was that we performed posterior capsular release for the cases with flexion contracture after the setting of the trial component, so we could not determine the influence of the change in PCO on the postoperative range of motion.

Secondly, the small number of cases is limiting, but the data is valuable because there are only a few cases of increased PCO in both condyles after TKA. The third limitation was that the change of joint line and overall knee alignment were not evaluated.

## Conclusions

In the present study, there was no correlation between the change in PCO and the extension gap. However, there was an inverse correlation between the flexion contracture and extension gap in cases with increased medial and lateral PCO. In such cases, we should create a larger extension gap by releasing the posterior capsule and performing a capsulectomy or manipulation to avoid postoperative flexion contracture.
